# De Novo Biosynthesis of *p*-Coumaric Acid in *E. coli* with a *trans*-Cinnamic Acid 4-Hydroxylase from the Amaryllidaceae Plant *Lycoris aurea*

**DOI:** 10.3390/molecules23123185

**Published:** 2018-12-03

**Authors:** Yikui Li, Jie Li, Binbin Qian, Li Cheng, Sheng Xu, Ren Wang

**Affiliations:** 1 Jiangsu Key Laboratory for the Research and Utilization of Plant Resources, Institute of Botany, Jiangsu Province and Chinese Academy of Sciences, Nanjing 210014, China; liyikui@cnbg.net (Y.L.); lj734820531@163.com (J.L.); qian_binbin01@163.com (B.Q.); chengli9075@163.com (L.C.); xusheng@cnbg.net (S.X.); 2 The Jiangsu Provincial Platform for Conservation and Utilization of Agricultural Germplasm, Nanjing 210014, China

**Keywords:** *p*-coumaric acid, *trans*-cinnamic acid 4-hydroxylase, *Lycoris aurea*, *Escherichia coli*, synthetic biology

## Abstract

*p*-Coumaric acid is a commercially available phenolcarboxylic acid with a great number of important applications in the nutraceutical, pharmaceutical, material and chemical industries. *p*-Coumaric acid has been biosynthesized in some engineered microbes, but the potential of the plant CYP450-involved biosynthetic route has not investigated in *Escherichia coli*. In the present study, a novel *trans*-cinnamic acid 4-hydroxylase (C4H) encoding the *Lau*C4H gene was isolated from *Lycoris aurea* (L’ Hér.) Herb via rapid amplification of cDNA ends. Then, *N*-terminal 28 amino acids of *Lau*C4H were characterized, for the subcellular localization, at the endoplasmic reticulum membrane in protoplasts of *Arabidopsis thaliana*. In *E. coli*, *Lau*C4H without the *N*-terminal membrane anchor region was functionally expressed when fused with the redox partner of *A. thaliana* cytochrome P450 enzyme (CYP450), and was verified to catalyze the *trans*-cinnamic acid to *p*-coumaric acid transformation by whole-cell bioconversion, HPLC detection and LC-MS analysis as well. Further, with phenylalanine ammonia-lyase 1 of *A. thaliana*, *p*-coumaric acid was de novo biosynthesized from glucose as the sole carbon source via the phenylalanine route in the recombinant *E. coli* cells. By regulating the level of intracellular NADPH, the production of *p*-coumaric acid was dramatically improved by 9.18-fold, and achieved with a titer of 156.09 μM in shake flasks. The recombinant cells harboring functional *Lau*C4H afforded a promising chassis for biological production of *p*-coumaric acid, even other derivatives, via a plant CYP450-involved pathway.

## 1. Introduction

*p*-Coumaric acid is a commercially available phenolcarboxylic acid with a great number of important applications in the nutraceutical, pharmaceutical, material and chemical industries. *p*-Coumaric acid possesses potent anti-oxidant, antibacterial and anti-inflammatory properties, and serves as a conventional precursor for the production of flavors and fragrances used in edible and daily chemical products. *p*-Coumaric acid is also a starting material for the preparation of environmentally degradable thermoplastics with liquid crystalline behavior [1]. Recently, *p*-coumaric acid has been found to have many novel bioactivities, such as antiproliferative effect [2], anxiolytic effect [3], a nephroprotective role [4], melanogenesis inhibition [5], and neuroprotective effects [6]. In addition, *p*-coumaric acid, as an upstream metabolite in the plant phenylpropanoid pathway, is a common precursor for biosynthesizing numerous derivatives, such as other phenylpropanoids [7,8], flavonoids [9,10], stilbenes [11] and anthocyanins [12].

**Figure 1:**
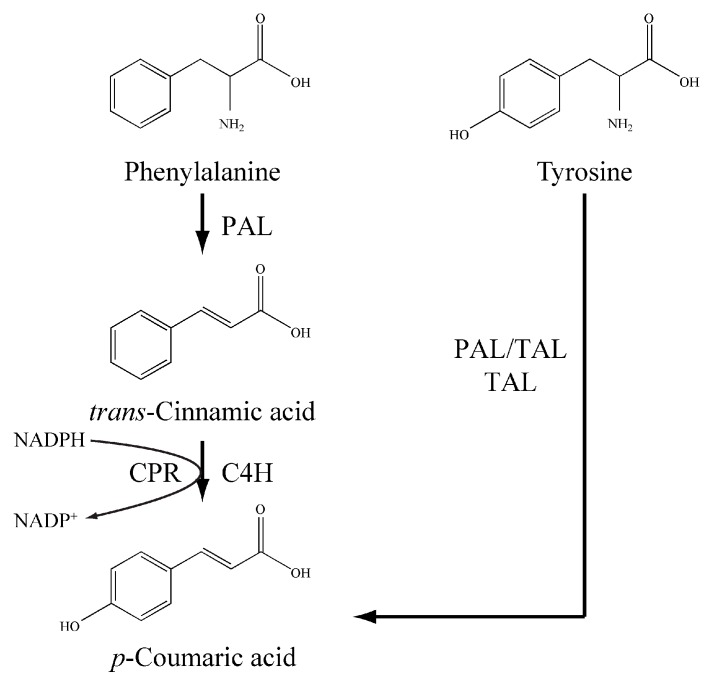
The biosynthetic routes of *p*-coumaric acid. The phenylalanine route (PAL-C4H) presents ubiquitously in plants and the tyrosine route (PAL/TAL and TAL) exists in some microbes.

*p*-Coumaric acid has been biosynthesized mainly from tyrosine in engineered microbes. *p*-Coumaric acid, as a component of lignin, is ubiquitously present in plants at a low concentration [13]. In plants, the biosynthesis of *p*-coumaric acid involves two biochemical processes: phenylalanine ammonia-lyase (PAL) firstly catalyzes the conversion of phenylalanine to *trans*-cinnamic acid, which is then hydroxylated at the *para* position under the action of *trans*-cinnamic acid 4-hydroxylase (C4H) (Figure 1) [14,15]. However, some PAL enzymes can accept tyrosine as an alternative substrate (PAL/TAL) and directly form *p*-coumaric acid from tyrosine without the intermediacy of *trans*-cinnamic acid (Figure 1) [16]. Also, there are some aromatic amino acid ammonia-lyase homolog enzymes specific for tyrosine deamination (TAL) (Figure 1) [17], so introducing a heterologous PAL/TAL or TAL, *p*-coumaric acid could be produced via the tyrosine route in recombinant cells such as *Escherichia coli* [18,19,20], *Saccharomyces cerevisiae* [21], *Streptomyces lividans* [22] and *Pseudomonas putida* [23].

In virtue of the plant biosynthetic route PAL-C4H, *p*-coumaric acid has been biosynthesized from phenylalanine only in engineered *S. cerevisiae* [18]. The enzyme C4H is a member of the CYP73A subfamily in cytochrome P450 enzymes (CYP450s) catalyzing a series of oxidation reactions with a CYP450 reductase as redox partner for supplying electrons from NADPH, and is supposed to commonly localize at the cytoplasmic side of endoplasmic reticulum (ER) membrane [14]. Given that prokaryotic microbes such as *E. coli* do not possess compartmentalized organelle(s), the functional expression of CYP450s is difficult [24,25]. Vannelli and his co-workers have functionally co-expressed the C4H and the CYP450 reductase from *Helianthus tuberosus* with a fungal PAL enzyme in *S. cerevisiae* cells, and produced *p*-coumaric acid from the central metabolite L-phenylalanine via the PAL-C4H route [18].

Though *p*-coumaric acid can be produced directly from tyrosine, it was necessary to investigate the potential of the plant CYP450-involved biosynthetic route from phenylalanine in *E. coli* which could to some extent expand the biosynthetic pathway of *p*-coumaric acid and offer an alternative approach to produce *p*-coumaric acid from phenylalanine as well as tyrosine. In the present study, based on our previous transcriptome data of *Lycoris aurea* (L’ Hér.) Herb [26], an ornamentally and medicinally important plant of the *Lycoris* genus of the Amaryllidaceae family, a novel C4H encoding gene was isolated from *L. aurea*, and designated as *Lau*C4H. Then *Lau*C4H was expressed truncatedly at the *N*-terminus in protoplasts of *A. thaliana* to identify the amino acids responsible for the subcellular localization. Moreover, in *E. coli*, *Lau*C4H without the N-terminal membrane anchor region was heterogeneously expressed for functional identification. PAL1 of *A. thaliana* was further introduced in the recombinant *E. coli* for *p*-coumaric acid de novo biosynthesis from glucose via the phenylalanine route. By regulating the level of intracellular NADPH, the production of *p*-coumaric acid was further increased by 9.18-fold, and a titer of 156.09 μM was achieved in shake flasks.

## 2. Results and Discussion

### 2.1. C4H Homology of L. aurea Transcriptome

Previously, de novo transcriptome sequencing has been performed to produce a comprehensive expressed sequence tag (EST) dataset for *L. aurea* using high-throughput sequencing technology [26]. The EST dataset of *L. aurea* provides a platform be critical in the speeding-up identification of a large number of related genes of secondary metabolite products. Further batch alignment results revealed that 226 contigs and unigenes were annotated to be responsible for the phenylpropanoid biosynthetic pathway. Of them, one unigene, namely unigene CL5217, showing high similarity with plant C4Hs was retrieved. Unigene CL5217 was 1794 bp with a predicted 1518 bp open reading frame (ORF), and was selected for further molecular cloning and functional characterization for the biosynthesis of *p*-coumaric acid.

### 2.2. Cloning of Full-length C4H Genes in L. aurea

**Figure 2:**
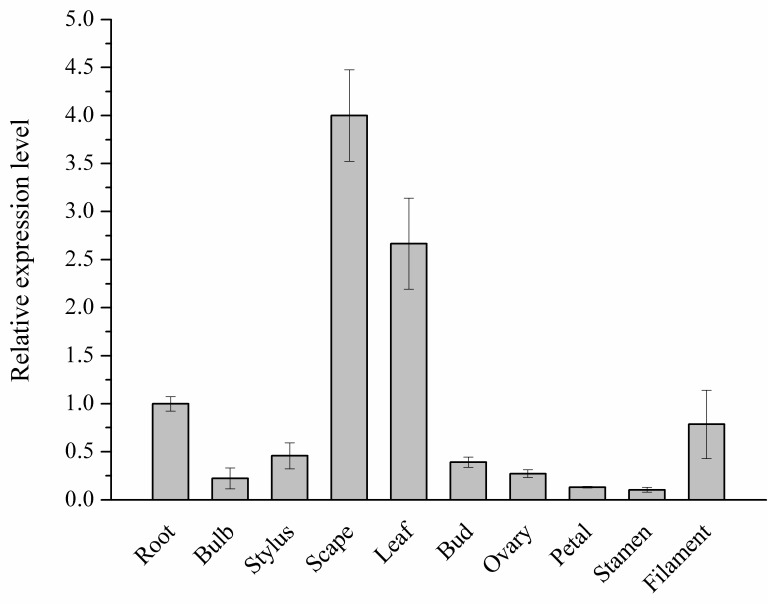
The abundance of *C4H* candidate gene (unigene CL5217) in various tissues of *L. aurea*. The values and error bars represent the mean ± standard error from three independent samples in three replicates per sample.

By quantitative real-time polymerase chain reaction (qRT-PCR) and rapid amplification of cDNA ends (RACE) approaches, a cDNA encoding C4H homology was isolated from *L. aurea*. Firstly the quantitative PCR primer pairs annealing at the 3′-terminus of the predicted ORF and 3′-untranslational region were designed and used to determine the abundance of the unigene CL5217 in various tissues of *L. aurea*. qRT-PCR results revealed that the level of unigene CL5217 was highest in scape (Figure 2).

**Figure A1:**
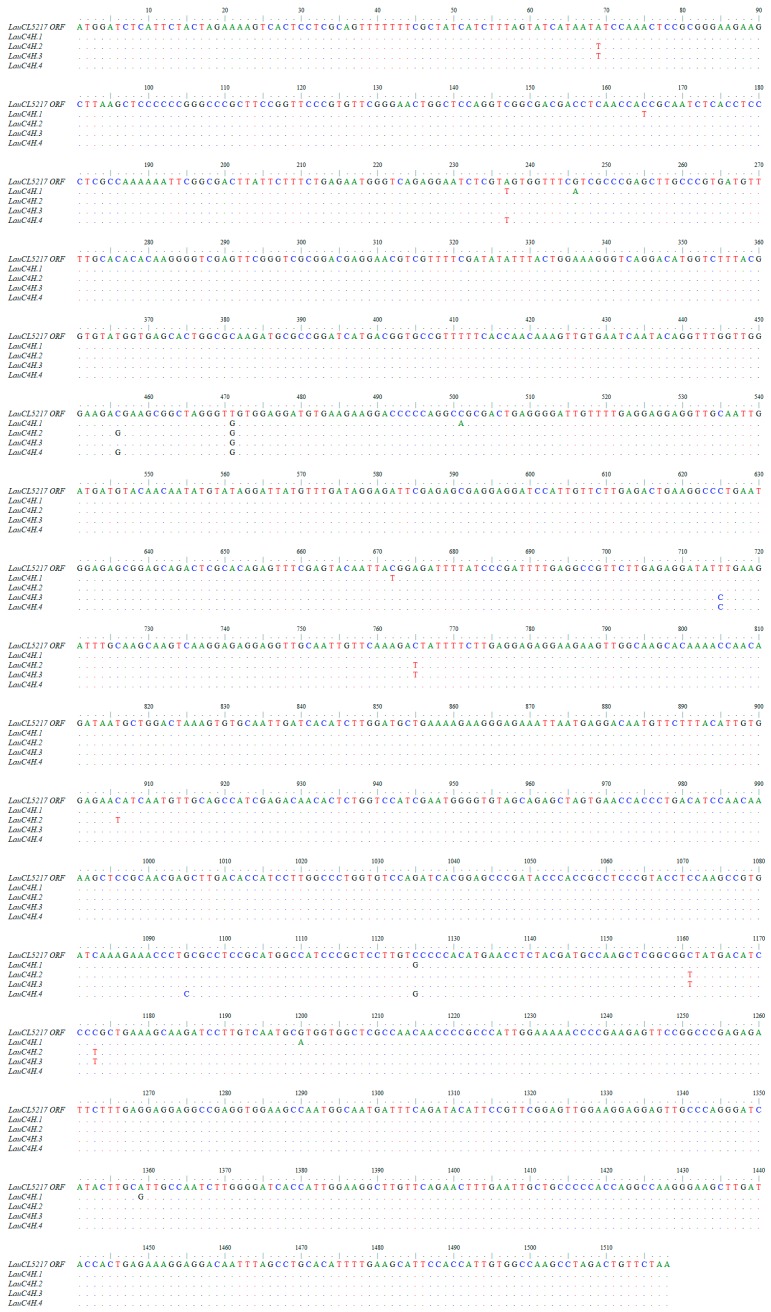
Coding sequence alignment of *Lau*C4H variants. Four unique variants of *Lau*C4H gene are aligned against the original sequence predicted by the de novo assembled transcriptome. Dots are identical bases.

**Figure A2:**
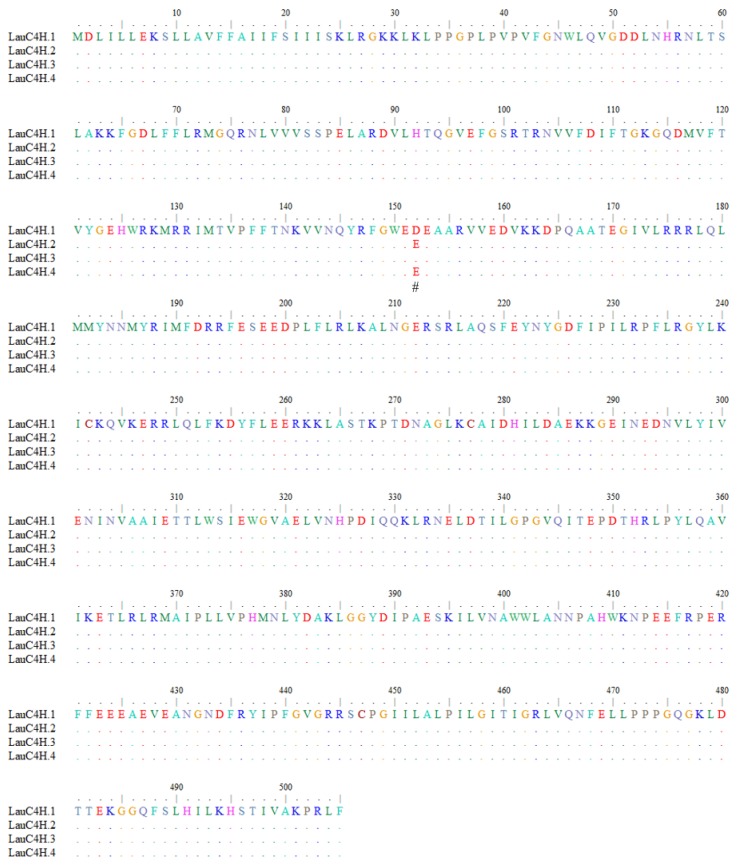
Protein sequence alignment of *Lau*C4H variants. Four unique variants of *Lau*C4H protein are aligned against the original sequence predicted by the de novo assembled transcriptome. Dots are identical residues.

Then, based on the sequence of unigene CL5217, the predicted ORF was cloned with the cDNA pool of scape as the template. Four gene variants were obtained with above 99% identity to each other at the nucleic acid level (Appendix Figure A1) referring to two protein variants at the amino acid level (Appendix Figure A2). Based on the genome size observed by flow cytometry, the plant material *L. aurea* used in this study is diploid (2n = 16) [27]. Therefore, a number of C4H paralogs were expected in the genome of *L. aurea*. Moreover, the plant used in this study was flowering and the bulb has been vegetatively propagated. For these reasons, the existence of multiple similar *C4H* transcripts was not surprising. This phenomenon was also reported in cloning the coding genes of norbelladine 4′-*O*-methyltransferase and *para-para’* C–C phenol coupling CYP450 in the Amaryllidaceae plant *Narcissus sp. aff. pseudonarcissus* [28,29]. The deduced *Lau*C4H protein (variant 1) had a predicted molecular mass of 58.19 kDa and pI of 9.04.

**Figure 3:**
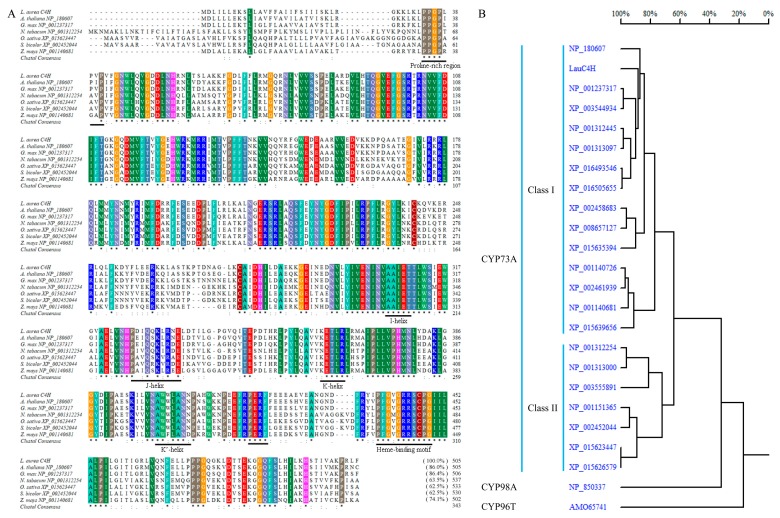
Sequence alignment (**A**) and homology analysis (**B**) of the full-length amino acid sequence of *Lau*C4H with other C4Hs reported in *A. thaliana* (NP_180607), *Glycine max* (NP_001237317, XP_003544934, XP_003555891), *Nicotiana tabacum* (NP_001312254, NP_001312445, NP_001313000, NP_001313097, XP_016493546, XP_016505655), *Oryza sativa* Japonica (XP_015623447, XP_015626579, XP_015635394, XP_015639656), *Sorghum bicolor* (XP_002452044, XP_002458683, XP_002461939) and *Zea mays* (NP_001140681, NP_001140726, NP_001151365, XP_008657127). GenBank accession numbers are indicated in parentheses. The threshold for shading in panel A was 100%. CYP98A3 (NP_850337) from *A. thaliana* and CYP96T1 (AMO65741) from *Narcissus* sp. *aff. pseudonarcissus* were additionally analyzed in panel B.

*Lau*C4H possessed all the diagnostic features of the primary structure for CYP450s as well as the CYP73A subfamily. A hydrophobic membrane-spanning region was predicted in the *N*-terminus of *Lau*C4H between amino acid residues 9 and 26 by TMpred online program (Swiss Institute of Bioinformatics (SIB), Lausanne, Switzerland) and SignalP 3.0 server [30]. Following the *N*-terminal transmembrane sequence, a proline-rich region (PPGPLPVP) was present (Figure 3A). A conserved heme-binding motif (PFGVGRRSCPG) was also found near the *C*-terminus of *Lau*C4H (Figure 3A). In addition, the “PERF” consensus sequence and some conserved helices such as I-helix (AAIET), J-helix (PDIQQKLRNE), K-helix (KETLR) and K’-helix (AWWLANN) were also identified in *Lau*C4H sequence (Figure 3A). In the homologous tree, plant C4Hs were grouped into two classes as that implied by the differential N-terminus and *C*-terminus in the amino acid sequence alignment (Figure 3A,B), and *Lau*C4H showed higher identity with the C4Hs of Class I than those of Class II (Figure 3B).

### 2.3. Subcellular Localization of LauC4H

**Figure 4:**
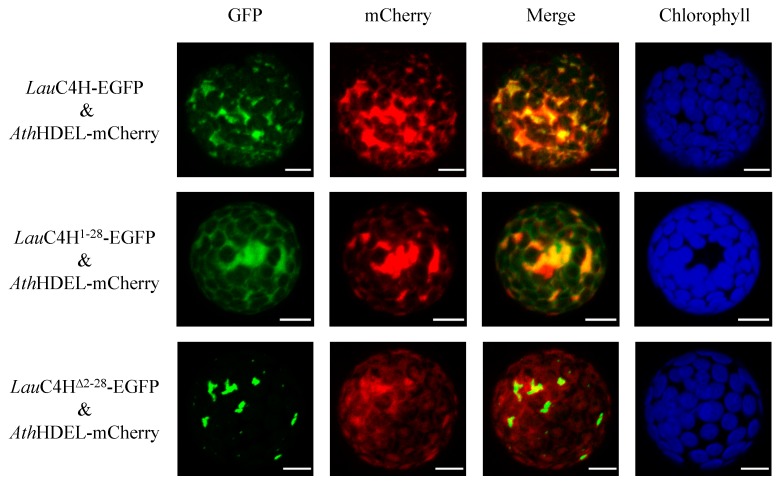
Subcellular localization of *Lau*C4H. “Green” panels show the fluorescence of *Lau*C4H variants fused with the enhanced green fluorescent protein (EGFP), “mCherry” panels indicate the fluorescence of endoplasmic reticulum marker (HDEL), and “Chlorophyll” panels indicate the auto-fluorescence of chloroplast. “Merged” panels represent the combined fluorescence from EGFP and mCherry. Bars = 10 μm.

**Figure 5:**
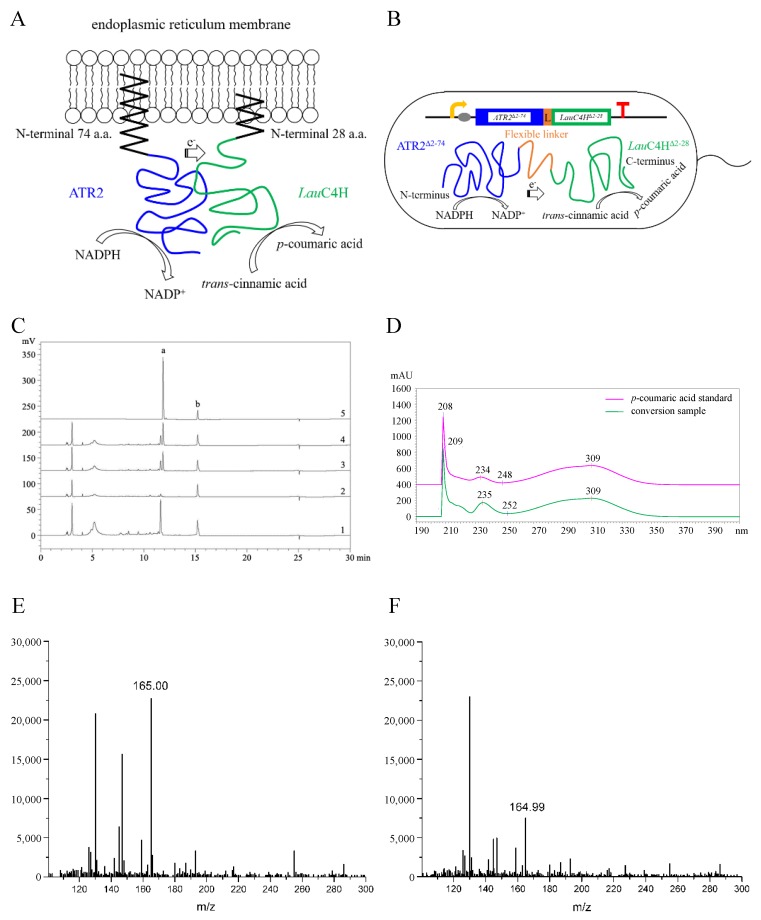
Functional identification of *Lau*C4H. (**A**) Diagram of endoplasmic reticulum co-localization of *Lau*C4H and *A. thaliana* cytochrome P450 reductase 2 (ATR2) in plant cells. (**B**) Heterologous fusion expression of *Lau*C4H and ATR2 via a flexible linker in *E. coli* without both N-terminal membrane anchor region. (**C**) HPLC analysis of reaction products from pET29a-ATR2^Δ2–74^ (1), pET29a (2), pET29a-ATR2^Δ2–74^*Lau*C4H.1^Δ2–28^ (3) and pET29a-ATR2^Δ2–74^*Lau*C4H.2^Δ2–28^ (4) using *trans*-cinnamic acid as the substrate. Standards (5) refer to HPLC analysis result of standard substance mixture of *p*-coumaric acid (a) and *trans*-cinnamic acid (b). (**D**) UV absorption spectra of the new product and the standard *p*-coumaric acid. (**E**,**F**) LC-MS identification of reaction products generated by recombinant *Lau*C4H.1 (**E**) and *Lau*C4H.2 (**F**).

Further, an investigation of the subcellular localization of *Lau*C4H in plant cells was carried out. *Lau*C4H was firstly predicted to localize in the ER using the WoLF PSORT program [31], and its N-terminal sequence was considered responsible for anchoring to the membrane using the online software TMpred (SIB) and SignalP 3.0 [30]. Then, the whole ORF and the truncated sequences encoding the *N*-terminal 28 amino acids (*Lau*C4H^1–28^) and the *Lau*C4H without the *N*-terminal 28 amino acids (*Lau*C4H^Δ2–28^), were fused with the enhanced green fluorescence protein (EGFP) gene, respectively. These fusion transgenes were transiently expressed in protoplasts prepared from the tender leaf of *A. thaliana*. Meanwhile, the fusion protein HDEL-mCherry with red fluorescence was used as the ER marker [32]. In *Arabidopsis* protoplasts containing *Lau*C4H-EGFP and HDEL-mCherry, the green fluorescence was present overlapping with the red fluorescence of the ER marker (Figure 4), showing a typical fluorescence pattern of ER localization. A similar pattern was also observed for the *Lau*C4H^1–28^-EGFP fusion protein overlapping with the ER marker, though there was a small portion of green fluorescence not overlapped with the red fluorescence (Figure 4). In *Arabidopsis* protoplasts containing *Lau*C4H^Δ2–28^-EGFP, the fluorescence pattern was distinctly different from those of *Lau*C4H-EGFP and *Lau*C4H^1–28^-EGFP, and there was scarcely any overlap between the green fluorescence and the red fluorescence of the ER marker (Figure 4). Thereby, our observation suggested that the *N*-terminal 28 amino acids spanning the hydrophobic transmembrane region was responsible for leading *Lau*C4H to localize in ER of plant cells. Previous studies showed that the CYP450 redox partners from *A. thaliana* and hybrid poplar were localized at the ER membrane of *Arabidopsis* protoplasts [33,34], those supported the notion that the *N*-terminus maybe confer *Lau*C4H to co-localize with the redox partner at the ER membrane and guide the electron transfer in plant cells (Figure 5A).

### 2.4. Functional Identification of LauC4H

Given that the oxygenation process of CYP450s requires a redox partner as electron supplier, the *A. thaliana* CYP450 redox partner ATR2 was employed for the functional exploration of *Lau*C4H in *E. coli*. Since there was no internal ER-like membrane in *E. coli*, both *Lau*C4H and ATR2 were expressed heterogenously without the *N*-terminal membrane anchor region, rather than in the full-length sequence, thus to avoid the so-called incompatibility between the membrane recognition signal of heterogenous proteins and the prokaryotic host [25]. In detail, the truncated ATR2 (ATR2^Δ2–74^) was fused in the N-terminus of truncated *Lau*C4H (*Lau*C4H^Δ2–28^) via a flexible octapeptide linker for electron supply (Figure 5B). The linker and the following proline-rich region in *Lau*C4H could orient optimally between ATR2 and *Lau*C4H [25,35]. When the recombinant *E. coli* cells harboring the chimeric ATR2^Δ2–74^-*Lau*C4H^Δ2–28^ protein were incubated with the substrate *trans*-cinnamic acid, a new product peak with the same retention time of *p*-coumaric acid was detected by HPLC analysis (Figure 5C). UV-Vis absorption spectra confirmed that the new product was identical to *p*-coumaric acid (Figure 5D).

The corresponding product was further examined using a liquid chromatography-mass spectrometry (LC-MS) in positive ion mode. LC-MS analysis of the targeted peak of the two LauC4H paralogs displayed the [M + H]^+^ ions at m/z 165 (Figure 5E,F), corresponding to the calculated molecular weight of *p*-coumaric acid (MW = 164.16). In contrast, no any visible peak with the same retention time of p-coumaric acid was observed in the conversion systems with ATR2^Δ2–74^ only as well as the empty vector pET29a, even though, with trans-cinnamic acid as the substrate. These results indicated that the new product synthesized with the chimeric ATR2^Δ2–74^-LauC4H^Δ2–28^ protein was *p*-coumaric acid. Thus, the LauC4H is an authentic trans-cinnamic acid 4-hydroxylase able to participate the conversion of trans-cinnamic acid into p-coumaric acid with the plant CYP450 redox partner (Figure 1).

### 2.5. p-Coumaric Acid De Novo Biosynthesis Using LauC4H in E. coli

**Figure A3:**
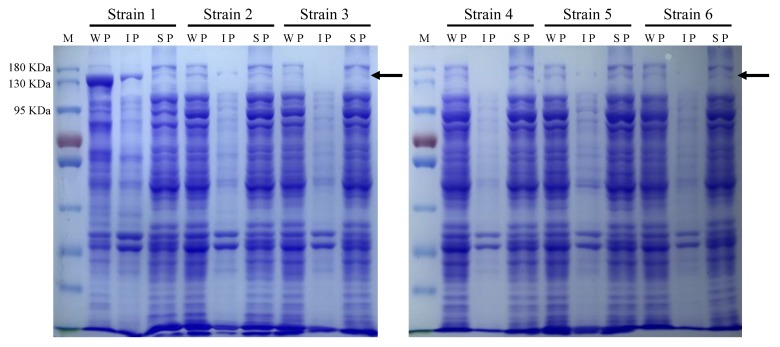
The expression level and solubility of the chimeric ATR2^Δ2–74^-*Lau*C4H^Δ2–28^ fusion protein in the *p*-coumaric acid producers. Strain 1, Ec/*Lau*C4H; Strain 2, Ec/*Lau*C4H-*Ath*PAL; Strain 3, Ec/*Lau*C4H-*Ath*PAL-sRNA; Strain 4, Ec/*Lau*C4H-*Ath*PAL-PntAB; Strain 5, Ec/*Lau*C4H-*Ath*PAL-anti(sthA); Strain 6, Ec/*Lau*C4H-*Ath*PAL-PntAB-anti(sthA). KDa, kilodalton; M, protein marker; WP, whole-cell proteins; IP, insoluble proteins; SP, soluble proteins. The arrows indicated the chimeric ATR2^Δ2–74^-*Lau*C4H^Δ2–28^ fusion protein.

For the application of *Lau*C4H to de novo biosynthesize *p*-coumaric acid, the *E. coli* cells harboring the functional chimera ATR2^Δ2–74^-*Lau*C4H^Δ2–28^ were cultured in mineral medium added with glucose as the sole carbon source. However, no *p*-coumaric acid was detected (Table 1), indicating that *E. coli* could not produce *trans*-cinnamic acid. When *A. thaliana* phenylalanine ammonia-lyase 1 (*Ath*PAL1) was further introduced to the *E. coli* cells mentioned above, *p*-coumaric acid could be produced and accumulated in the medium of the recombinant cells with ATR2^Δ2–74^-*Lau*C4H^Δ2–28^ and *Ath*PAL1 (Table 1). Therefore, the plant CYP450-involved *p*-coumaric acid biosynthesis pathway has been successfully established in procaryotic *E. coli*. Moreover, within a duration of 42 h induction with IPTG, the recombinant *E. coli* produced 17.22 μM of *p*-coumaric acid. The expression level and solubility of the chimeric ATR2^Δ2–74^-*Lau*C4H^Δ2–28^ fusion protein were detected in Ec/*Lau*C4H and Ec/*Lau*C4H-*Ath*PAL (Figure A3). As shown in Figure A3, the expression level of the chimeric ATR2^Δ2–74^-*Lau*C4H^Δ2–28^ fusion protein in Ec/*Lau*C4H was more than that in Ec/*Lau*C4H-*Ath*PAL. However, the level of the soluble ATR2^Δ2–74^-*Lau*C4H^Δ2–28^ fusion protein was comparable between Ec/*Lau*C4H and Ec/*Lau*C4H-*Ath*PAL. Comparably, *E. coli* cells only with *Ath*PAL1 or PAL/TAL from the yeast *Rhodotorula glutinis* (*Rgl*PAL/TAL) [18] were also cultured as controls. In cells only with *Ath*PAL1, *trans*-cinnamic acid rather than *p*-coumaric acid was detected in the medium (Table 1). In cells only with *Rgl*PAL/TAL, both *trans*-cinnamic acid and *p*-coumaric acid were accumulated, of which the concentration was 91.94 μM and 46.09 μM, respectively (Table 1). Notably, there was 342.82 μM of *trans*-cinnamic acid detected in the medium of the recombinant cells with ATR2^Δ2–74^-*Lau*C4H^Δ2–28^ and *Ath*PAL1 (Table 1).

### 2.6. p-Coumaric Acid Production Improved by Intracellular NADPH Regulation

**Figure 6:**
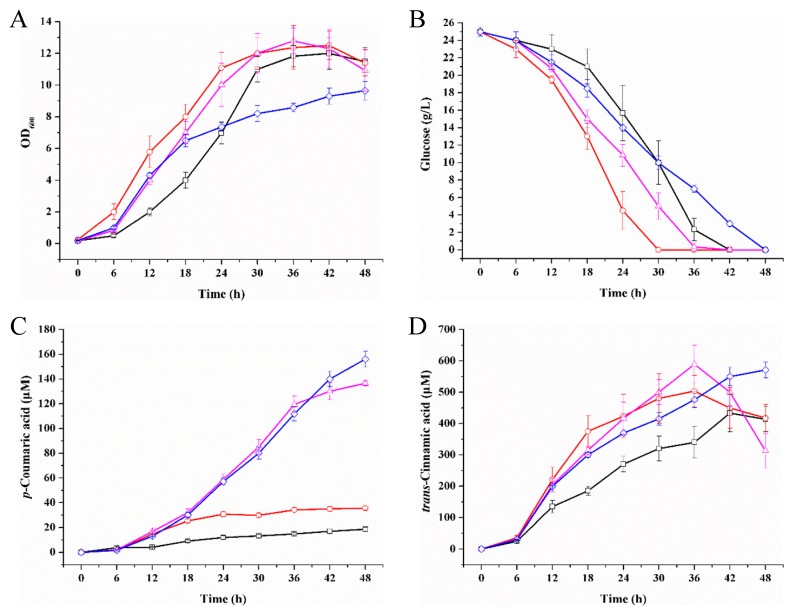
Regulation of intracellular NADPH to improve the *p*-coumaric acid production. (**A**) cell growth; (**B**) glucose utilization; (**C**) *p*-coumaric acid biosynthesis; (**D**) *trans*-cinnamic acid accumulation. The *p*-coumaric acid producers were Ec/*Lau*C4H-*Ath*PAL-sRNA (black square), Ec/*Lau*C4H-*Ath*PAL-anti(sthA) (red circle), Ec/*Lau*C4H-*Ath*PAL-PntAB (magenta triangle), and Ec/*Lau*C4H-*Ath*PAL-PntAB-anti(sthA) (blue diamond). The error bars represent the standard error of the mean from three independent experiments of each strain.

The biochemical process of *Lau*C4H catalysis of the conversion of *trans*-cinnamic acid into *p*-coumaric acid needs two electrons per mole of substrate. ATR2 as a CYP450 redox partner is NADPH-dependent [33,34]. Since we observed that about 20-fold the substrate of *Lau*C4H still existed in the medium of strain Ec/*Lau*C4H-*Ath*PAL (Table 1), we were assuming that the output of *p*-coumaric acid was subjected to the level of intracellular NADPH (Figure 1). For testing this assumption, we attempted to elevate the level of intracellular NADPH. Using synthetic small regulatory RNA (srRNA) anti(sthA) [36] to specific repression of the translation of the soluble transhydrogenase SthA (also referred to as UdhA) [37], the conversion of NADPH to NADH may be down-regulated when treated with anti(sthA) so that the content of NADPH would be relatively enhanced. As shown in Figure 6, the introduction of the srRNA anti(sthA) accelerated cell growth and glucose utilization, and resulted in about 2-fold increase of the *p*-coumaric acid production. In addition, we also pursued overexpression of the membrane-bound transhydrogenase PntAB catalyzing the NADH to NADPH conversion [37]. When the overexpression of five-copy *pntAB* driven by a T7 promoter took place, the production of *p*-coumaric acid was dramatically improved by 7.93-fold up to 136.53 μM (Figure 6C). Both over-expressed PntAB and srRNA anti(sthA) resulted in a 9.18-fold increase, along with relative lower biomass and slower glucose consumption. Thus over-expressed PntAB and srRNA anti(sthA) played a synergetically positive effect on the de novo biosynthetic production of *Lau*C4H-mediated *p*-coumaric acid. The expression level and solubility of the chimeric ATR2^Δ2–74^-*Lau*C4H^Δ2–28^ fusion protein in those *p*-coumaric acid producers were comparable (Figure A3). Under such circumstance, there was a considerable amount of *trans*-cinnamic acid yet in the medium, indicating that the chimera ATR2^Δ2–74^-*Lau*C4H^Δ2–28^ was involved in a rate-limiting step for the formation of *p*-coumaric acid besides NADPH. Subsequently, there should be more approaches to be tested for improving the output of *p*-coumaric acid in *E. coli*. For example, the turnover of *Lau*C4H could be enhanced either by increasing the expression level of the chimera or by modulating the spatial structure of the chimera using modularized bioengineering tools in synthetic biology.

## 3. Materials and Methods

### 3.1. Plant Materials and Chemicals

The *Lycoris aurea* plants used in this study were collected from Nanjing Botanical Garden Mem. Sun Yat-Sen (Nanjing, China), and were about three-year old when the flowers bloom unless otherwise stated. *A. thaliana* wild-type (Columbia ecotype) plants used in this study were grown at 22 °C for four weeks after germination. Chemicals and reagents used in this study were purchased from either Sigma-Aldrich (St. Louis, MO, USA) or Sangon Biotech (Shanghai, China).

### 3.2. RNA Extraction and Isolation of LauC4H Genes

Total RNA from different tissues of *L. aurea* were extracted using the RNAprep pure Plant Kit (TIANGEN, Beijing, China). The cDNA pool was then synthesized by PrimerScript^TM^ RT reagent Kit (TaKaRa, Dalian, China). To quantify the unigene CL5217 expression in different tissues, quantitative real-time polymerase chain reaction (qRT-PCR) was performed using AceQ^®^ qPCR SYBR^®^ Green Master Mix (High ROX Premixed) (Vazyme Biotech, Nanjing, China) with the gene *LauTIP41* as internal reference [38]. To obtain desired sequences, 5′- and 3′- rapid-amplification of cDNA ends (RACE) were carried out using the SMARTer^TM^ RACE cDNA Amplification Kit (Clontech Laboratories, Inc., Mountain View, CA, USA) according to the manufacturer’s manual. The full-length cDNA were verified by re-amplification of the open reading frame (ORF) using forward primer *Lau*C4H-ORF-PF and reverse primer *Lau*C4H-ORF-PR. PCR protocols were as follows: one cycle of 5 min at 98 °C; 30 cycles with a denaturing time of 45 s at 95 °C, an annealing time of 45 s at 56 °C, and an elongation time of 90 s at 72 °C; and a final elongation step of 10 min at 72 °C. The PCR products of expected size were excised from the EtBr-stained 1% (*w*/*v*) agarose gels, purified using the DNA Gel Extraction Kit (BioTeke Corporation, Beijing, China) and ligated into the pMD19-T vector (TaKaRa) for sequencing. Each of the transcripts was obtained from at least three different monoclones. The primers used in this experiment are listed in Appendix Table A1.

### 3.3. Sequence Aanalysis of LauC4H

The open reading frames (ORFs) in the obtained cDNA sequences were searched and the correspondingly encoded protein sequences were exported on NCBI ORFfinder webpage (https://www.ncbi.nlm.nih.gov/orffinder/) (Bethesda, MD, USA). Then, homology search of the predicted protein were performed using regular blastp on NCBI BLAST webpage (https://blast.ncbi.nlm.nih.gov/Blast.cgi) (Bethesda, MD, USA). Molecular mass and isoelectric point of translated proteins were predicted on the SIB Bioinformatics Resource Portal ExPASy (https://www.expasy.org/) (Lausanne, Switzerland). The subcellular localization in plant cells was predicted using the WoLF PSORT program (https://wolfpsort.hgc.jp/) [31]. The prediction of signal sequence and transmembrane sequence was carried out using the SignalP 3.0 Server (http://www.cbs.dtu.dk/services/SignalP-3.0/) [30] and the TMpred online program (https://embnet.vital-it.ch/software/TMPRED_form.html) (SIB). Amino acid sequences of full-length C4Hs were aligned and analyzed by the BioEdit Sequence Alignment Editor Version 7.1.3.0 [39] and the ClustalW program (SIB), respectively. The homologous tree was constructed via the software DNAMAN Version 8 (Lynnon Corporation, San Ramon, CA, USA).

### 3.4. Subcellular Localization Analysis of LauC4H

To determine the subcellular localization of *Lau*C4H, enhanced green fluorescent protein (EGFP) was in frame fused to the C-terminus of the *Lau*C4H protein sequence under the control of the dual cauliflower mosaic virus (CaMV) 35S promoter in the pAN580 vector. The plasmid pAN580 was digested by *Nco*I (TaKaRa, Dalian, China). The DNA fragments without termination codon encoding full-length *Lau*C4H, *N*-terminal 28 amino acids of *Lau*C4H (*Lau*C4H^1–28^) and N-terminal truncated *Lau*C4H (*Lau*C4H^Δ2–28^) were prepared with primer pairs pAN580-*Lau*C4H-PF and EGFP-*Lau*C4H-PR, primer pairs pAN580-*Lau*C4H-PF and EGFP-*Lau*C4H(N28)-PR, and primer pairs pAN580-*Lau*C4H(ΔN28)-PF and EGFP-*Lau*C4H-PR, respectively. The DNA fragments were assembled to linear pAN580 by ClonExpress One Step Cloning Kit (Vazyme Biotech) to create pDual35S::*Lau*C4H-EGFP, pDual35S::*Lau*C4H^1–28^-EGFP and pDual35S::*Lau*C4H^Δ2–28^-EGFP. Primers used to make these constructs were designed according to the product manual and are listed in Appendix Table A1. The well-established fluorescent protein marker mCherry-HDEL [32] was used for the indication of the endoplasmic reticulum (ER). All transient expression constructs were transformed separately into *Arabidopsis* protoplasts with the mCherry-HDEL construct according to the method [40]. The transformed samples were incubated for 16–18 h before examination. Fluorescent images were observed by a laser scanning confocal microscope using a Zeiss LSM780 camera (Carl Zeiss Microscopy GmbH, Jena, Germany).

### 3.5. Heterologous Expression of LauC4H in Escherichia coli

*Escherichia coli* Top10 was used as the host for molecular cloning. For gene expression in *E. coli*, Novagen^®^’s pET29a vector (EMD Millipore Corporation, Darmstadt, Germany) was used and digested by *Nde*I and *Xho*I (TaKaRa). The DNA fragment encoding *N*-terminal truncated *Lau*C4H (*Lau*C4H^Δ2–28^) were amplified with primer pairs 29a*Nde*I-*Lau*C4H(ΔN28)-PF and 29a*Xho*I-*Lau*C4H-PR. The DNA encoding the cytochrome P450 redox partner ATR2 without N-terminal 74 amino acids (ATR2^Δ2–74^) was obtained with the primer pairs 29a*Nde*I-ATR2(ΔN74)-PF and 29a*Xho*I-ATR2-PR from *A. thaliana* cDNA pool as the template. The DNA fragments was assembled to linear pET29a by ClonExpress One Step Cloning Kit (Vazyme Biotech) to create pET29a-*Lau*C4H^Δ2–28^ and pET29a-ATR2^Δ2–74^. To express *Lau*C4H functionally in *E. coli*, the truncated ATR2 with a short flexible octapeptide (GSTSSGSG) was prepared with the primer pairs 29a*Nde*I-ATR2(ΔN74)-PF and *Lau*C4H(ΔN28)ATR2-PR, and was in frame fused to the N-terminus of *Lau*C4H^Δ2–28^ into the *Nde*I site of pET29a-*Lau*C4H^Δ2–28^ by ClonExpress One Step Cloning Kit (Vazyme Biotech) to create pET29a-ATR2^Δ2–74^*Lau*C4H^Δ2–28^. Primers used to make these constructs are listed in Appendix Table A1. *E. coli* Rosetta (DE3) (Vazyme Biotech) was used as the host for protein expression. Freshly transformed *E. coli* Rosetta (DE3) cells harboring pET29a-derived plasmids were grown at 37 °C and 220 rpm in M9Y minimal medium [41] with 20 g L^−1^ glucose as carbon source to A_600_ of 0.6–0.8, and then were induced with 0.1 mM isopropyl-β-D-thiogalactoside (IPTG) at 28 °C overnight.

### 3.6. Bioconversion of LauC4H with trans-Cinnamic Acid

Whole-cell bioconversion strategy was adopted to identify the function of *Lau*C4H. 50 mL cell cultures were harvested by centrifugation at 8000 rpm and 4 °C for 5 min, washed twice and re-suspended in 40 mL nitrogen-free M9Y mineral medium. The re-suspended cells were added 20 g L^−1^ glucose and 100 μM *trans*-cinnamic acid, and incubated at 28 °C with a stirring rate of 250 rpm for the whole-cell bioconversion. Samples were taken with an interval of 12 h, an equal volume of methanol was added to terminate the reaction and the samples were then subjected to HPLC analysis.

### 3.7. p-Coumaric Acid De Novo Biosynthesis in E. coli

To in vivo biosynthesize the substrate of *Lau*C4H in *E. coli* cells, the *AthPAL1* gene encoding *A. thaliana* phenylalanine ammonia lyase 1 was overexpressed in pACYC184 vector [42,43] under a trc promoter induced by IPTG. For gene overexpression, pACYC184 was digested by *Nco*I and *Eco*RI (TaKaRa, Dalian, China) to get the plasmid skeleton with p15A origin of replication and tetracycline-resistant gene. Primer pairs 184-trc-lacO-PF and BBa_B0034-lacO-PR were annealed and elongated, and then purified to obtain the DNA fragment trcO-RBS containing the core-trc promoter (−10 box and −35 box), lacI binding site and the BioBrick ribosome binding site (RBS) BBa_B0034 chosen from the MIT Registry of Standard Biological Parts (http://parts.igem.org/Main_Page) (The International Genetically Engineered Machine (iGEM), Cambridge, MA, USA). Primer pairs BBa_B0034-BBa_B0015-PF and 184-BBa_B0015-PR were annealed and elongated, and then purified to obtain the DNA fragment BBa_B0015 containing the BioBrick terminator BBa_B0015 also chosen from the MIT Registry of Standard Biological Parts (iGEM, Cambridge, MA, USA). The three fragments, plasmid skeleton, trcO-RBS and BBa_B0015 terminator, sharing the overlap one by one, were assembled together by ClonExpress Ultra One Step Cloning Kit (Vazyme Biotech). The new plasmid was named as p15A-trcO3415. Then, the *AthPAL1* gene was amplified from the *A. thaliana* cDNA pool with primers BBa_B0034-*AthPAL1*-PF and BBa_B0015-*AthPAL1*-PR and assembled into the linear p15A-trcO3415 by *Nde*I restriction endonuclease (TaKaRa) via ClonExpress One Step Cloning Kit (Vazyme Biotech) to create p15A-trcO*Ath*PAL1. Then p15A-trcO*Ath*PAL1 along with the plasmid pET29a-ATR2^Δ2–74^*Lau*C4H^Δ2–28^ were transformed into *E. coli* Rosetta (DE3) cells to obtain the recombinant strain Ec/*Lau*C4H-*Ath*PAL. The empty vector p15A-trcO3415 along with the plasmid pET29a-ATR2^Δ2–74^*Lau*C4H^Δ2–28^ were transformed into *E. coli* Rosetta (DE3) cells to obtain the recombinant strain Ec/*Lau*C4H. The plasmid p15A-trcO*Ath*PAL1 along with the empty vector pET29a were transformed into *E. coli* Rosetta (DE3) cells to obtain the recombinant strain Ec/*Ath*PAL. The primers involved in this experiment are summarized in Appendix Table A1.

**Figure A4:**
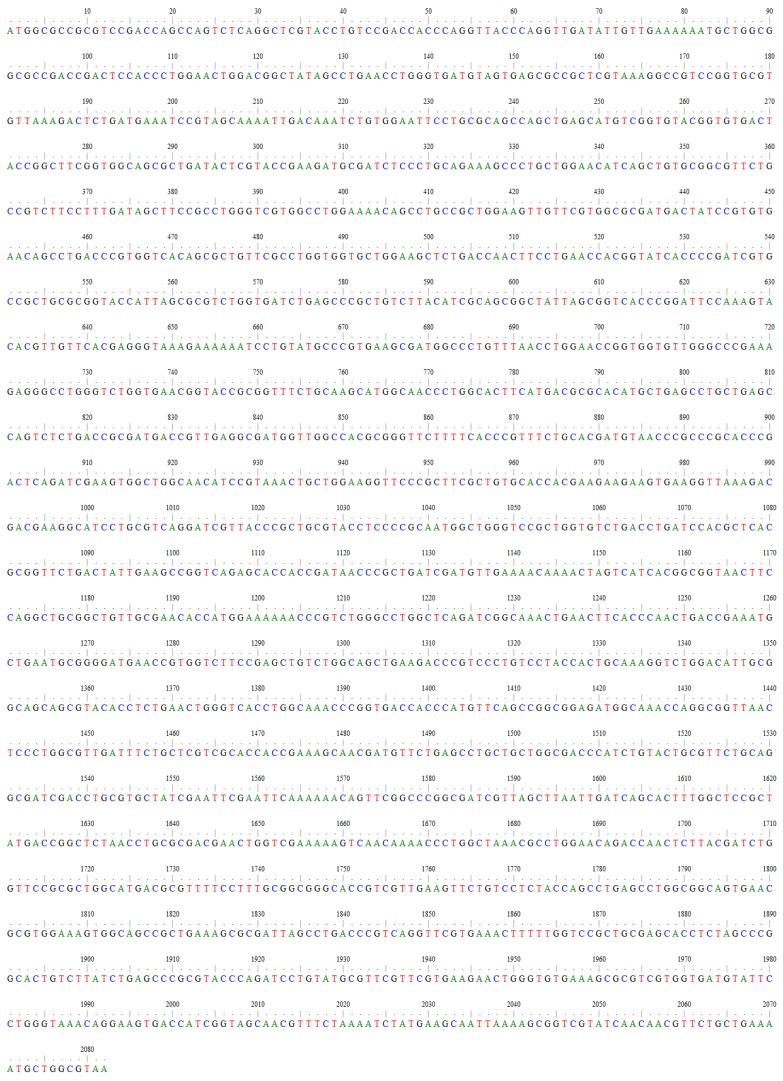
The codon-optimized DNA sequence of the yeast *Rhodotorula glutinis* PAL/TAL.

The DNA sequence encoding the PAL/TAL from the yeast *Rhodotorula glutinis* [18] was optimized according to the codon preference of *E. coli*, and synthesized on the clone vector pUC57 to obtain pUC57-Opt*Rgl*PAL/TAL plasmid by Sangon Biotech (Shanghai) Co., Ltd (Shanghai, China). The *RglPAL/TAL* gene was amplified from the plasmid pUC57-Opt*Rgl*PAL/TAL with primers BBa_B0034-*RglPAL/TAL*-PF and BBa_B0015-*RglPAL/TAL*-PR and assembled into the linear p15A-trcO3415 by *Nde*I restriction endonuclease (TaKaRa) via ClonExpress One Step Cloning Kit (Vazyme Biotech) to create p15A-trcO*Rgl*PAL/TAL. Then p15A-trcO*Rgl*PAL/TAL plasmid along with the empty vector pET29a were transformed into *E. coli* Rosetta (DE3) cells to obtain the recombinant strain Ec/*Rgl*PAL(TAL). The primers are summarized in Appendix Table A1 and the optimized DNA sequence of *Rgl*PAL/TAL was shown in Appendix A Figure A4.

A single clone of the recombinant *E. coli* strains was incubated in 3 mL LB medium for 24 h. Then 1 mL of the cell cultures was collected, the supernatant was discarded, and the cell pellet was transferred into 50 mL fermentation medium and grown at 37 °C and 200 rpm. The fermentation medium contains 100 mM 3-(*N*-morpholino)propanesulfonic acid (MOPS), 28.71 mM K_2_HPO_4_, 25.72 mM KH_2_PO_4_, 26.50 mM (NH_4_)_2_HPO_4_, 10.00 mM citric acid, 5.00 mM MgSO_4_, 1 μg L^−1^ vitamin B1, 0.5 μg L^−1^ biotin, 166.67 mM glucose, 0.02 mM phenol red, and 10 mL trace element solution. The trace element solution was prepared in 100 mM HCl with 36.00 mM FeSO_4_, 7.82 mM ZnSO_4_, 4.00 mM CuSO_4_, 2.00 mM MnSO_4_, 0.60 mM Na_2_B_4_O_7_, 13.60 mM CaCl_2_, and 0.08 mM (NH_4_)_6_MO_7_O_24_. The cells were induced with 0.1 mM IPTG at 30 °C when grown to A_600_ of 0.6–0.8. The pH in the medium was maintained at about 7.0 by adding ammonium hydroxide aperiodically. Samples were taken with an interval of 6 h and analyzed by HPLC.

### 3.8. Intracellular NADPH Regulation

For down-regulation of *EcoSthA* gene, the synthetic sRNA-based strategy [36] was applied. The plasmid skeleton with pSC101 origin of replication and spectinomycin-resistant gene was obtained from pCL1920 [44] as previously described [45]. The P_R_-MicC fragment including the constitutive P_R_ promoter and the MicC scaffold was amplified from the wild-type *E. coli* MG1655 genome with primer pairs BBa_R0051-MicC-PF and BBa_B0015-MicC-PR. The BBa_B0015 terminator was amplified from the plasmid p15A-trcO3415 with primer pairs BBa_B0015-PF and BBa_B0015-PR. The P_R_-MicC-BBa_B0015 fragment was amplified with primer pairs pCL-BBa_R0051-PF and pCL-BBa_B0015-PR from both of P_R_-MicC fragment and BBa_B0015 terminator as the template. Then, the plasmid skeleton and the P_R_-MicC-BBa_B0015 fragment were assembled by ClonExpress One Step Cloning Kit (Vazyme Biotech) to construct the new plasmid pSC101-sRNA for synthetic sRNA production. Subsequently, the *N*-terminal 24 bp of *EcoSthA* gene was obtained by annealing and elongation with primer pairs anti(*sthA*)-PF and anti(*sthA*)-PR, and assembled into the linear pSC101-sRNA by *Nsi*I restriction endonuclease (TaKaRa) via ClonExpress One Step Cloning Kit (Vazyme Biotech) to create pSC101-anti(sthA).

For appropriate overexpression of NAD(P) transhydrogenase, the two genes *EcopntAB* were overexpressed with their native ribosome binding sites under a constitutive T7 promoter and transcriptionally terminated by the BioBrick terminator BBa_B1006 also chosen from the MIT Registry of Standard Biological Parts (iGEM, Cambridge, MA, USA). The *EcopntAB* DNA fragment was amplified from the wild-type *E. coli* MG1655 genome with primer pairs pCL-T7-*pntA*-PF and pCL-BBa_B1006-*pntB*-PR, and assembled into the *Kpn*I (TaKaRa) digested pSC101-sRNA and pSC101-anti(sthA) via ClonExpress One Step Cloning Kit (Vazyme Biotech) to create pSC101-OE(pntAB) and pSC101-anti(sthA)-OE(pntAB), respectively. Then pSC101-sRNA, pSC101-anti(sthA), pSC101-OE(pntAB) and pSC101-anti(sthA)-OE(pntAB), respectively, was transformed into strain Ec/*Lau*C4H-*Ath*PAL cells to obtain the recombinant strain Ec/*Lau*C4H-*Ath*PAL-sRNA, Ec/*Lau*C4H-*Ath*PAL-anti(sthA), Ec/*Lau*C4H-*Ath*PAL-PntAB, and Ec/*Lau*C4H-*Ath*PAL-PntAB-anti(sthA).

### 3.9. Protein Detection

Cells induced for 12 h were harvested by centrifugation at 8000 rpm for 5 min at 4 °C, washed twice with TNG buffer (20 mM Tris-HCl pH 7.9, 0.5 M NaCl, 10% Glycerol), and re-suspended in the same buffer containing 1 mM phenylmethylsulfonyl fluoride (PMSF). The suspended cells were sonicated on ice-bath followed by centrifugation at 12,000 rpm for 30 min at 4 °C. The supernatant was transferred out, and the sediment was re-suspended in the PMSF-contained TNG buffer. Proteins were assessed by SDS-PAGE.

### 3.10. High Performance Liquid Chromatography (HPLC) Analysis

All samples taken from the cultures were centrifuged at 12,000 rpm for 2 min and the supernatants were filtered through a 0.22 μm polytetrafluorethylene (PTFE) filter and analyzed by HPLC (Shimadzu LC-20A, Kyoto, Japan) with a reverse phase Shimadzu InertSustain C18 column (5 μm, 4.6 mm × 250 mm) and a Shimadzu SPD-M20A photodiode array detector. The mobile phase used was a gradient of solvent A (H_2_O containing 1.3% acetic acid) and solvent B (100% acetonitrile) applied as following time procedure: 0–20 min, 10–100% B linear; 20–20.5 min, 100%–10% linear; 20.5–30 min, 10% B isocratic. The flow rate was set at 1.0 mL·min^−1^, and the injection volume was 10 μL. The column was maintained at 35 °C, and the eluted compounds were monitored at 309 nm for *p*-coumaric acid and at 274 nm for *trans*-cinnamic acid respectively. The concentration of *trans*-cinnamic acid and *p*-coumaric acid were quantified by fitting the peak area with a standard curve (R^2^ > 0.999) of the corresponding standard.

## 4. Conclusions

In the present study, *p*-coumaric acid was de novo produced via the plant CYP450-involved biosynthetic route in *Escherichia coli*. Firstly, a novel CYP73A from the Amaryllidaceae plant *Lycoris aurea* was cloned based on the transcriptome data, of which the *N*-terminal 28 amino acids were characterized responsible for localizing at the endoplasmic reticulum membrane in protoplasts of *Arabidopsis thaliana*. Then, *Lau*C4H was expressed functionally in *E. coli* when fused with the CYP450 redox partner from *A. thaliana*, and shown to catalyze the conversion of *trans*-cinnamic acid into *p*-coumaric acid. Further, *p*-coumaric acid was de novo biosynthesized via introducing a phenylalanine ammonia-lyase from *A. thaliana* into the recombinant *E. coli* cells. The production of *p*-coumaric acid was dramatically improved via regulation of the intracellular NADPH level of the constructed cell factory. The producer reported herein afforded a promising chassis for the biosynthesis of *p*-coumaric acid-derived molecules with great application value.

## Figures and Tables

**Table 1:** *p*-Coumaric acid de novo biosynthesis using *Lau*C4H in *E. coli*. ^1^

Strain	Biomass (OD_600_)	Glucose Utilization (g/L)	*trans*-Cinnamic Acid (μM)	*p*-Coumaric Acid (μM)
Ec/*Lau*C4H	5.56 ± 0.72	13.50 ± 0.72	ND ^2^	ND ^2^
Ec/*Ath*PAL	8.92 ± 0.85	24.50 ± 0.65	241.32 ± 13.24	ND ^2^
Ec/*Rgl*PAL(TAl)	9.73 ± 1.24	25.08 ± 0.75	91.94 ± 6.32	46.09 ± 2.96
Ec/*Lau*C4H-*Ath*PAL	7.66 ± 0.83	24.78 ± 1.26	342.82 ± 15.80	17.22 ± 1.23

^1^ The data represent the mean ± the standard error from three independent experiments of each strain. ^2^ ND, not detected.

## Appendix A

**Table A1:** Oligonucleotides used in this study. ^1^

Name	Sequence (5′ to 3′)
RT-PCR	
RT-*Lau*C4H-PF	AAGGGAAGCTTGATACCACTGAGAA
RT-*Lau*C4H-PR	GACAAATTAGAACAGTCTAGGCTTGGC
RT-*LauTIP41*-PF	GCAACCATCCAAAGTTTAACTGCT
RT-*LauTIP41*-PR	AATGTGCAAGCAGGGCTAGTAA
cDNA cloning	
*Lau*C4H-ORF-PF	ATCCTCCTCCTCCGACGAAATG
*Lau*C4H-ORF-PR	AGAGTACATTGCATGGGTAATAAGGAG
*Lau*C4H-5′RACE-PR	GAACTCGACCCCTTGTGTGTGC
*Lau*C4H-3′RACE-PF	GGCTATGACATCCCCGCTGA
Subcellular localization	
pAN580-*Lau*C4H-PF	AGGACCGGTCCCGGGGGATCCATGGATCTCATTCTACTAGAAAAGTCACTC
EGFP-*Lau*C4H-PR	TCCTCGCCCTTGCTCACCATGAACAGTCTAGGCTTGGCCACAATG
EGFP-*Lau*C4H(N28)-PR	TCCTCGCCCTTGCTCACCATCCCGCGGAGTTTGGATATTATGAT
pAN580-*Lau*C4H(ΔN28)-PF	AGGACCGGTCCCGGGGGATCCATGAAGAAGCTTAAGCTCCCCCCGG
Functional expression	
29a*Nde*I-*Lau*C4H(ΔN28)-PF	AACTTTAAGAAGGAGATATACATATGAAGAAGCTTAAGCTCCCCCCGG
29a*Xho*I-*Lau*C4H-PR	AGTGGTGGTGGTGGTGGTGCTCGAGTTAGAACAGTCTAGGCTTGGCCACAATG
29a*Nde*I-ATR2(ΔN74)-PF	AACTTTAAGAAGGAGATATACATATGTCCGGTTCTGGGAATTCAAAA
29a*Xho*I-ATR2-PR	AGTGGTGGTGGTGGTGGTGCTCGAGTGAGTGTGTGGCTTCAATAGTTTCG
*Lau*C4H(ΔN28)ATR2-PR	CCGGGGGGAGCTTAAGCTTCTTCATACCAGAACCAGAAGAGGTAGAACCCCATACATCTCTAAGATATCTTCCACTCGTTTG
De novo biosynthesis	
184-trc-lacO-PF	GCCTTGCGTATAATATTTGCCCATGGTTGACAATTAATCATCCGGCTCGTATAATGTGTGGAATTGTGAGCGG
BBa_B0034-lacO-PR	CATATGTATTTCTCCTCTTTAGATCTGGAATTGTTATCCGCTCACAATTCCACACATTATAC
BBa_B0034-BBa_B0015-PF	ATCTAAAGAGGAGAAATACATATGCTCGAGTAACCAGGCATCAAATAAAACGAAAGGCTCAGTCGAAAGACTGGGCCTTTCGTTTTATCTGTTGTTTGTCGGTGAACGCTCTCTACTA
184-BBa_B0015-PR	GCCCGCCTGATGAATGCTCATCCGGAATTCTATAAACGCAGAAAGGCCCACCCGAAGGTGAGCCAGTGTGACTCTAGTAGAGAGCGTTCACCGACAAA
BBa_B0034-*Ath*PAL1-PF	ATCTAAAGAGGAGAAATACATATGGAGATTAACGGGGCACACAA
BBa_B0015-*Ath*PAL1-PR	TTATTTGATGCCTGGTTACTCGAGACTTTATGGTAAGAAAAAAACAGAGGAC
BBa_B0034-*Rgl*PAL/TAL-PF	ATCTAAAGAGGAGAAATACATATGGCGCCGCGTCCGACCAGCCA
BBa_B0015-*Rgl*PAL/TAL-PR	TTATTTGATGCCTGGTTACTCGAGTTACGCCAGCATTTTCAGCAGA
NADPH regulation	
BBa_R0051-MicC-PF	TATTTTACCTCTGGCGGTGATAATGGTTGCATGCATTTTCTGTTGGGCCATTGCATTGC
BBa_B0015-MicC-PR	CGTTTTATTTGATGCCTGGCTCGAGAAAAAAAGCCCGGACGACTGTTC
BBa_B0015-PF	GCCAGGCATCAAATAAAACG
BBa_B0015-PR	TATAAACGCAGAAAGGCCCAC
pCL-BBa_R0051-PF	ACCCGTCTTACTGTCGGGAATTCTAACACCGTGCGTGTTGACTATTTTACCTCTGGCGGTGAT
pCL-BBa_B0015-PR	ACGTTGTAAAACGACGGCCAGTGGTACCTATAAACGCAGAAAGGCCCAC
anti(*sthA*)-PF	CCTCTGGCGGTGATAATGGTTGCATCGTAATCGTAGGAATGTGGCAT
anti(*sthA*)-PR	ATGCAATGGCCCAACAGAAAATGCCACATTCCTACGATTACGAT
pCL-T7-*pntA*-PF	GGGCCTTTCTGCGTTTATAGGTACCAAATTAATACGACTCACTATAGGGGAATCTAGAGGGAATATCATGCGAATTGGCATACC
pCL-BBa_B1006-*pntB*-PR	TTGTAAAACGACGGCCAGTGGATCCAAAAAAAACCCCGCCCTGTCAGGGGCGGGGTTTTTTTTTAAGCTTGGGTTACAGAGCTTTCAGGATTGC

^1^ The sequences with single underline and double underline are the homologous arms for one-step cloning and the flexible linker for the protein fusion, respectively.
